# Analysis of *CYP1B1* Polymorphisms in Lung Cancer Patients Using Novel, Quick and Easy Methods Based on CAPS and ACRS-PCR Techniques

**DOI:** 10.3390/ijms25126676

**Published:** 2024-06-18

**Authors:** Adam Dąbrowski, Maciej Nowicki, Aleksandra Budzyńska, Jakub Suchodolski, Rafał Ogórek, Mariusz Chabowski, Katarzyna Przywara

**Affiliations:** 1 Laboratory of Molecular Diagnostics “Bio-Genetik” NZOZ, 50-525 Wrocław, Poland; laboratorium.biogenetik@gmail.com; 2 Department of Surgery, 4th Military Teaching Hospital, 53-114 Wroclaw, Poland; m.r.nowicki@wp.pl (M.N.); mariusz.chabowski@gmail.com (M.C.); 3 Department of Mycology and Genetics, Faculty of Biological Sciences, University of Wrocław, 51-148 Wrocław, Poland; 310568@uwr.edu.pl (A.B.); jakub.suchodolski@uwr.edu.pl (J.S.); rafal.ogorek@uwr.edu.pl (R.O.); 4 Department of Nursing and Obstetrics, Division of Anesthesiological and Surgical Nursing, Faculty of Health Science, Wroclaw Medical University, 50-556 Wroclaw, Poland; 5 Department of Clinical Surgical Sciences, Faculty of Medicine, Wroclaw University of Science and Technology, 51-377 Wroclaw, Poland

**Keywords:** CYP1B1, polymorphisms, lung cancer, CAPS-PCR, ACRS-PCR

## Abstract

Within the sequence of the *CYP1B1* gene, more than 50 polymorphisms, resulting from single-nucleotide polymorphisms (SNPs), have been described. Some of them play an important role as specific genetic markers in the process of carcinogenesis and for therapeutic purposes. In this publication, we present methods we have developed that enable the specific and unambiguous identification of four polymorphisms that result in amino acid changes: c. 142C > G, c. 355G > T, c. 1294C > G, and c. 1358A > G. Our studies are based on cleaved amplified polymorphic sequences (CAPSs) and artificially created restriction site (ACRS) PCR techniques; therefore, they require only basic laboratory equipment and low financial outlays. Utilizing the described methods allows for the reduction of research time and cost, and the minimization of errors. Their effectiveness and efficiency depend on the careful design of appropriate primers and the precise selection of suitable restriction enzymes. As a result, further confirmation by sequencing is not necessary. Using the developed method, we examined 63 patients diagnosed with lung cancer and observed a 1.5 to 2.1 times higher frequency of the analyzed single-nucleotide polymorphisms compared to the frequency in the European population.

## 1. Introduction

Cytochrome P450 (CYP450) is found in every class of living organism, including *Archaea*, and as organisms have evolved, its functionality has increased [1]. Its main role is detoxification, and in the human body, it occurs in almost every cell type, but it achieves the greatest activity in the cells of the small intestine, liver, and adrenal medulla [2]. CYP450 enzymes are characterized by low substrate specificity. They participate in hydroxylation, e.g., steroid hormones, bile acids, prostaglandins, or xenobiotics, including drugs, pesticides, tobacco smoke, and food additives [3]. In terms of oncology, a very interesting member of the CYP450 superfamily is the enzyme CYP1B1, which has been placed in a list of cancer antigen prioritization for targeted therapies by the National Cancer Institute [4]. The *CYP1B1* gene is located on chromosome 2p22.2 and consists of 12,000 base pairs divided into three exons and two introns. The mature transcript contains over 5000 base pairs and encodes a protein of 543 amino acids [5]. Unlike most CYPs, its expression is low in normal tissues; however, it is often overexpressed in cancer [6]. Like all cytochromes P450, it catalyzes oxidation reactions. Its main substrates are 17β-estradiol and polycyclic aromatic hydrocarbons (PAHs), which become carcinogens and can cause double-stranded DNA breaks [6,7]. Within the sequence of the *CYP1B1* gene, more than 50 polymorphisms resulting from SNPs have been described [8]. The best-known polymorphisms that result in amino acid changes include c. 142C > G (R48G), c. 355G > T (A119S), c. 1294C > G (L432V), c. 1358A > G (N453S) [7]. There are many scientific reports indicating a link between the presence of *CYP1B1* polymorphisms and the risk of lung cancer. They focus primarily on the c. 1294C > G, c. 1358A > G, and c. 355G > T isoforms [9,10,11,12,13,14,15]. Unfortunately, due to heterogeneous groups, the results are not unanimous.

*CYP1B1* polymorphisms play an important role as specific genetic markers in the process of carcinogenesis and for therapeutic purposes. Therefore, in this publication, we have developed fast, specific, and cost-effective methods for identifying c. 142C > G, c. 355G > T, c. 1294C > G, and c. 1358A > G polymorphisms using CAPS and ACRS-PCR techniques. In addition, using these methods, we examined 63 patients diagnosed with lung cancer. We subjected the obtained results to statistical analysis, considering histopathological diagnosis and cancer stage assessment.

## 2. Results

### 2.1. Diagnosis of Polymorphism in the CYP1B1 Gene

#### 2.1.1. c. 142C > G

**Figure 1:**
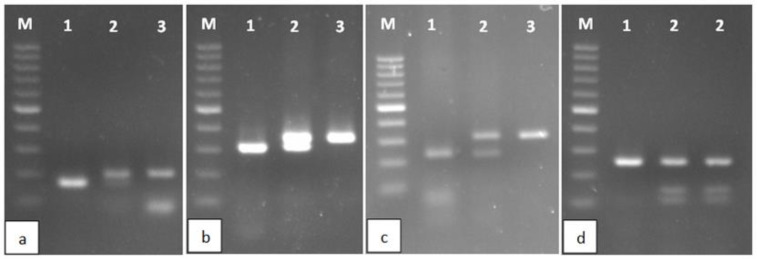
Restriction enzyme digestion products characteristic of polymorphisms: c. 142C > G (**a**), c. 355G > T (**b**), c. 1294C > G (**c**), and c. 1358A > G (**d**). M, mass ladder (100–1000 bp; contrast band: 500 bp); 1, wild-type gene (unmutated homozygote); 2, presence of a mutation in one allele (mutated heterozygote); 3, presence of mutations in both alleles (mutated homozygote).

In the case of this polymorphism, we observe a substitution of cytosine base for guanine [C/G]. In order to amplify the appropriate DNA fragment, appropriately constructed primers were used that changed the amplicon fragment beyond the mutation locus (Table 1). Consequently, the use of the restriction enzyme *Hpa*II resulted in the cleavage of the wild-type gene fragment at only one site, yielding 171 and 33 bp products. In contrast, the allele with the mutation remained undigested (204 bp) (Table 2, Figure 1).

#### 2.1.2. c. 355G > T

The *CYP1B1* c. 355G > T polymorphism was identified by digesting the amplified 364 bp gene fragment with *Nae*I restriction enzyme. This endonuclease recognizes a sequence at a potential mutation site. Therefore, strand scission occurred only in the wild type, producing 290 and 74 bp products. The mutant remained uncut (364 bp) (Table 2, Figure 1).

#### 2.1.3. c. 1294C > G

The amplified 310 bp gene fragment was subjected to the restriction enzyme *Oli*I, which recognizes sequences characteristic of the wild form of the gene. As a result, in the case of the L423 form, two products were obtained: 228 and 82 bp. In the presence of the c.1294C > G mutation, the product was not digested and remained at 310 bp (Table 2, Figure 1).

#### 2.1.4. c. 1358A > G

After amplification, a product of 324 bp was obtained. The *Mwo*I enzyme recognizes the 5′-GCNNNNNNNGC-3′ sequence. In the amplified fragment of the wild form of the gene, this sequence is present in two places, while in the mutated form, there is an additional place within the mutation itself. Thus, treatment of the amplicone with the *Mwo*I enzyme resulted in products of 36, 35, and 253 bp in the case of the wild-type allele and 36, 35, 108, and 145 bp in the presence of a nucleotide change (Table 2).

Overall information on the histopathological diagnosis, tumor staging, gender, age, smoking status, and genotypes of individual polymorphisms of all 63 examined patients is attached as Table S1 (Supplementary Materials).

### 2.2. Frequency of Occurrence of Individual Polymorphisms in the Analyzed Population of Lung Cancer Patients

**Figure 2:**
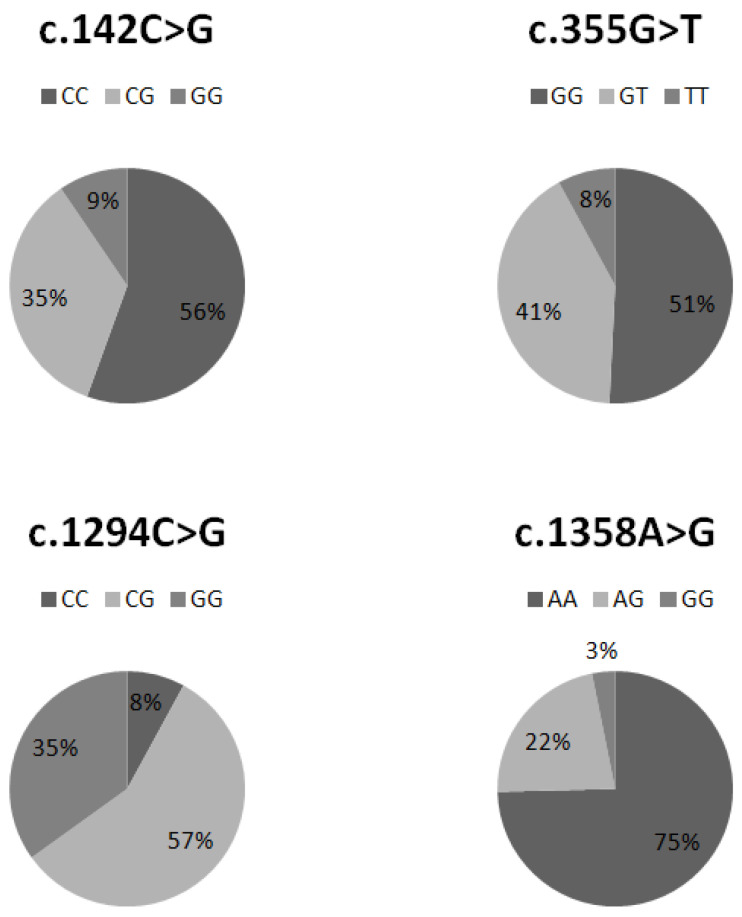
Diagrams showing the percentage distribution of individual genotypes of the tested polymorphisms in the analyzed population.

The percentage distribution of individual genotypes in the c. 142C > G, c. 355G > T, 1294C > G, and c. 1358A > G polymorphisms is illustrated in Figure 2.

### 2.3. Statistical Analysis between the Presence of SNPs and the Type and Stage of Cancer

During the study, we observed that the identified SNPs did not correlate with either the type or progression of the tumor disease. Each SNP was statistically insignificant when using the χ^2^ test at α = 0.01 (Table S2, Supplementary Materials). Thus, we conclude that neither SNP nor the size of the primary tumor, involvement of lymph nodes, or distant metastasis (TNM staging) affects the development of specific tumor types (glandular, squamous, or small-cell carcinoma).

## 3. Discussion

Lung cancer ranks first in terms of morbidity and mortality from malignant tumors in the world. It is the second most commonly diagnosed cancer in men following prostate cancer, and in women following breast cancer [16]. In 2018, the Global Cancer Observatory (GLOBOCAN) recorded over 2 million new cases of lung cancer and 1.8 million deaths caused by it [16]. Such results are influenced, among others, by the increase in urbanization, environmental pollution (mainly by PAHs), but, above all, by active and passive smoking [17]. Benzo[a]pyrene, the main component of tobacco smoke and environmental pollutants, is activated with the use of cytochrome P450 enzymes [18]. The main role in this process is played by the CYP1A1 and CYP1B1 enzymes, which convert PAHs into an intermediate form—epoxides—which are then subjected to the action of epoxide hydrolase, which modifies them into reactive mutagens [18]. The influence of polymorphisms in the *CYP1B1* gene in this process is not fully understood, but it is suggested that they increase the process of activation of mutagenic compounds [7].

The mutations we study are directly related to the formation of cancers conditioned by the influence of environmental factors, such as smoking, environmental pollution from combustion products of low-quality coal, petroleum hydrocarbons and plastics, contact with organic solvents, and the use of steroid hormones. The methods used in the study enable the determination of c. 142C > G, c. 355G > T, c. 1294C > G, and c. 1358A > G polymorphisms in the *CYP1B1* gene. They are based on the amplification of *CYP1B1* gene fragments and their digestion with appropriate restriction enzymes. The products are visualized using standard horizontal electrophoresis, allowing for unambiguous test result determination. Therefore, each of the diagnostic stages requires only basic laboratory equipment and low financial outlays. This way of obtaining results does not require further confirmation by sequencing the DNA of each patient [19]. The effectiveness and efficiency of the research presented by us is based on the careful design of appropriate primers and the precise selection of suitable restriction enzymes. Some of our restricted products are less than 40 bp in size (Table 2); therefore, they may appear faint in typical agarose gel. However, it should be noted that the rest of the restriction cleavage products from the same amplicon are sufficiently large and apparently differentiated depending on the genotype. Thus, the difficulty in reading small bands does not affect the diagnostic effectiveness.

Germline mutations are the basis of hereditary lung cancers [20]. The *CYP1B1* gene polymorphism can be homozygous or heterozygous. The presented tests allow us to determine whether a given patient has a mutation in one or two copies of the gene. This information may impact the course of the disease or treatment decisions. Studies suggest that patients who carry both mutated copies of the gene are characterized by more severe symptoms and worse response to treatment then heterozygotes [20]. Interestingly, the isoforms of this enzyme may also have a pharmacogenetic aspect. It has been observed that CYP1B1 isoforms can determine the effectiveness of treatment with, among others, taxanes or daunorubicin [21,22,23,24]. It must be noted that CYP1B1 does not metabolize these drugs.

According to the National Institutes of Health, the frequency of SNPs resulting in the c. 142C > G (rs10012), c. 355G > T (rs1056827), c. 1294C > G (rs1056836), and c. 1358A > G (rs1800440) polymorphisms in the European population is ~24, ~29, ~43, and ~17%, respectively. In our study group of 63 patients with lung cancer, we noted a higher frequency of the analyzed mutations, equal to approximately 44, 49, 92, and 25%, which corresponds to an increase of approximately 1.8, 1.7, 2.1, and 1.5 times, respectively. The most significant increase was observed in the c. 1294C > G polymorphism. This observation coincides with the conclusion presented in a meta-analysis from 2014, which showed a correlation between the occurrence of the L432V polymorphism and the development of lung cancer in the Caucasian, Asian, and African American populations [25]. It has been observed that individuals with the wild-type genotype (CC) exhibit higher levels of *CYP1B1* gene expression compared to GG homozygotes [26]. Exposure to tobacco smoke or environmental pollution implies that individuals with the mutated genotype are unable to effectively remove toxins from cells, contributing to DNA damage and the accumulation of other mutations [26].

In conclusion, the methods presented by us are fast, accurate, and unambiguous. ACRS-PCR and CAPS-PCR are very sensitive and specific methods [27]. Their use allows for the testing time to be shortened, costs to be reduced, and the possibility of errors to be minimized. Fast and effective diagnosis of *CYP1B1* polymorphisms increases the chances of quick implementation of effective therapy and good prognosis. In addition, due to the pharmacogenetic significance of individual polymorphisms, it may determine the choice of introducing effective chemotherapy.

## 4. Materials and Methods

### 4.1. Clinical Samples

The research material was obtained from patients at the 4th Military Hospital with a Polyclinic in Wrocław, Poland. The study material was blood collected on EDTA medium from 63 patients with confirmed lung cancer. Blood samples were taken just before the surgical removal of the cancerous lesion. The research group consisted of 37 women and 26 men. The percentage of female patients diagnosed with adenocarcinoma, squamous cell carcinoma, and small-cell carcinoma were ~46, ~35, and ~19%, respectively. In the case of male patients, it was ~54, ~34.5, and ~11.5%. A total of 57 out of 63 examined patients were in the age group of 60–80 years. Three patients were under 60 years of age and only two were over 80. Detailed information regarding gender, age, and histopathological diagnosis is presented in the Supplementary Materials (Table S1). All participants of the study were informed about the course of the scientific experiment and gave their written consent to participate in it.

### 4.2. DNA Isolation

An Xpure™ Blood mini kit (A&A Biotechnology, Gdansk, Poland) was used to isolate DNA from lymphocyte fractions from blood samples. The concentration of DNA was determined using a spectrophotometer GeneQuant II RNA/DNA Calculator (Pharmacia Biotech, Cambridge, UK). 

### 4.3. Determination of Polymorphism in CYP1B1 Gene

In order to minimize the risk of DNA contamination and obtaining incorrect results, the DNA isolation procedure and its further processing related to the PCR reaction were carried out at various stations dedicated to specific procedures. Every batch of analysis was performed in the presence of control DNA with the mutation and a negative control with water instead of genetic material. Each sample had a final volume of 50 μL, consisting of 80–120 ng of genomic DNA and 0.3 μM of each primer. The amplification was conducted under the following cycling conditions: 94 °C for 10 min, followed by 40 cycles at 94 °C for 30 s, annealing for 30 s (for c.142C > G, c.1294C > G, c.1358A > G genes—58 °C, for c.355G > T—55 °C), and 72 °C for 30 s, with a final extension at 72 °C for 5 min. CAPS and ACRS PCR methods were used to identify four selected polymorphisms in the *CYP1B1* gene [27]. Four separate PCR amplifications were performed for each sample using PCR Mix Plus (A&A Biotechnology, Gdansk, Poland) and a T100 Thermal cycler (Bio-Rad, Hercules, CA, USA). Appropriate primer pairs were designed for each PCR reaction, based on the current *CYP1B1* gene sequence available in the GenBank database of the National Center for Biotechnology Information (NM_000104.4). The primers allowed the amplification of *CYP1B1* gene fragments that contained restriction enzyme recognition sites. Then, 15 µL of the amplicon was digested with restriction enzymes (Thermo Fisher Scientific, Waltham, MA, USA; Table 1). The received products were separated in TBE buffer using 2% agarose (EURx, Gdansk, Poland) with SimplySafeTM (EURx, Gdansk, Poland) and visualized under ultraviolet light using Gel Doc^TM^ EZ Imager (Bio-Rad, Hercules, CA, USA).

#### 4.3.1. c. 142C > G 

The ACRS-PCR method was used to identify the c. 142C > G polymorphism. Appropriately selected primers (Table 1) made it possible to modify the amplicon in order to create a recognition site for the *Hpa*II enzyme (Table 2).

#### 4.3.2. c. 355G > T, c. 1294C > G and c. 1358A > G

The CAPS PCR method was used to identify three polymorphisms in the *CYP1B1* gene: c. 355G > T, c. 1294C > G, and c. 1358A > G. All of these mutations are the result of the substitution of nitrogenous bases (Table 1). After amplification of the DNA fragment containing the sought mutation, restriction digestion of the resulting amplicon was performed. For this purpose, the restriction enzymes *Nae*I, *Oli*I, and *Mwo*I were used, respectively, for the above-mentioned mutations (Table 2). 

### 4.4. Statistical Analysis

In order to establish whether SNPs correlate with tumor type and progression, statistical significance was determined using the χ^2^ test at α = 0.01 (Microsoft Excel 2010).

## Figures and Tables

**Table 1:** Oligonucleotides used for amplification (**A**—nucleotide change for restriction purposes).

Polymorphism	Primer Sequences 5′ → 3′	Amplicon Size (bp)
R48G c.142C > G	F: GTGTCACGCCTTCTCCTCTC R: TGGCCACGCAAACGGGCC**A**GG	204
A119S c.355G > T	F: ACGCTCCTGCTACTCCTGTC R: CCTTCCAGTGCTCCGAGTAG	364
L432V c.1294C > G	F: CAGGCAGAATTGGATCAGGT R: CTCTGCTGGTCAGGTCCTTG	310
N453S c.1358A > G	F: TGTCCTGGCCTTCCTTTATG R: GCCAGGATGGAGATGAAGAG	324

**Table 2:** Cleavage sites of restriction enzymes used in this study and size of the expected products after restriction. UIUPAC codes: **S**—C/G, **R**—A/G (↓—restriction enzyme cleavage site).

Polymorphism	Fragment of the Gene after Amplification	Product Size (bp)		
R48G c.142C > G	*Hpa*II CCGG GTGTCACGCCTTCTCCTCTCTGTCCCCAGCATGGGCACCAGCCTCAGCCCGAACGACCCTTGGCCGC**↓**TAAACCCGCTGTCCATCCAGCAGACCACGCTCCTGCTACTCCTGTCGGTGCTGGCCACTGTGCATGTGGGCCAGCGGCTGCTGAGGCAACGGAGGCGGCAGCTC**S**GGTCCGCGCCCCCTGGCCCGTTTGCGTGGCCA	CC	CG	GG
		171 33	204 171 33	204
A119S c.355G > T	*Nae*I GCCGGC ACGCTCCTGCTACTCCTGTCGGTGCTGGCCACTGTGCATGTGGGCCAGCGGCTGCTGAGGCAACGGAGGCGGCAGCTCCGGTCCGCGCCCCCGGGCCCGTTTGCGTGGCCACTGATCGGAAACGCGGCGGCGGTGGGCCAGGCGGCTCACCTCTCGTTCGCTCGCCTGGCGCGGCGCTACGGCGACGTTTTCCAGATCCGCCTGGGCAGCTGCCCCATAGTGGTGCTGAATGGCGAGCGCGCCATCCACCAGGCCCTGGTGCAGCAGGGCTCGGCGCC**↓**G**R**CCTTCGCCTCCTTCCGTGTGGTGTCCGGCGGCCGCAGCATGGCTTTCGGCCACTACTCGGAGCACTGGAAGG	GG	GT	TT
		290 74	364 290 74	364
L432V c.1294C > G	*Oli*I CACNNNNGTG CAGGCAGAATTGGATCAGGTCGTGGGGAGGGACCGTCTGCCTTGTATGGGTGACCAGCCCAACCTGCCCTATGTCCTGGCCTTCCTTTATGAAGCCATGCGCTTCTCCAGCTTTGTGCCTGTCACTATTCCTCATGCCACCACTGCCAACACCTCTGTCTTGGGCTACCACATTCCCAAGGACACTGTGGTTTTTGTCAACCAGTGGTCTGTGAATCATGACCCA**S**T**↓**GAAGTGGCCTAACCCGGAGAACTTTGATCCAGCTCGATTCTTGGACAAGGATGGCCTCATCAGCAAGGACCTGACCAGCAGAG	CC	CG	GG
		228 82	310 228 82	310
N453S c.1358A > G	*Mwo*I GCNNNNNNNGC TGTCCTGGCCTTCCTTTATGAAGCCATGCGCTTCTC**↓**CAGCTTTGTGCCTGTCACTATTCCTCATGCCACCA**↓**CTGCCAACACCTCTGTCTTGGGCTACCACATTCCCAAGGACACTGTGGTTTTTGTCAACCAGTGGTCTGTGAATCATGACCCACTGAAGTGGCCTAACCCGGAGAACTTTGATCCAGCTCGATTCTTGGACAAGGACGGCCTCAT**↓**CA**R**CAAGGACCTGACCAGCAGAGTGATGATTTTTTCAGTGGGCAAAAGGCGGTGCATTGGCGAAGAACTTTCTAAGATGCAGCTTTTTCTCTTCATCTCCATCCTGGC	AA	AG	GG
		253 36 35	253 145 108 36 35	145 108 36 35

## Data Availability

Data are available within the article and its Supplementary Materials.

## Supplementary Materials

The following supporting information can be downloaded at: https://www.mdpi.com/article/10.3390/ijms25126676/s1.
