# Supplementary material for: Analysis of CYP1B1 Polymorphisms in Lung Cancer Patients Using Novel, Quick and Easy Methods Based on CAPS and ACRS-PCR Techniques
Source: Int J Mol Sci. 2024 Jun 18;25(12):6676. doi: 10.3390/ijms25126676 (PMC11203417; doi:10.3390/ijms25126676)
Supplement: Supplementary file 1 [file ijms-25-06676-s001.zip › ijms-3023528-supplementary.pdf]

Table S1 General information on 63 examined patients, including: gender, age, tumor staging, smoking status, genotype of tested polymorphisms and histopathological diagnosis.

No.	Gender	Age	TNM staging	Number of pack-years	*CYP1B1* polymorphism				Histopathological diagnosis
					c.142C>G	c. 355G>T	c. 1294C >G	c. 1358A >G	
1	F	74	T2aN0M0	20	CC	GT	GG	AA	Adenocarcinoma
2	M	71	T1bN0M0	10	CG	GG	GG	AA	
3	F	51	T2aN0M1b	100	CC	GT	GG	AA	
4	F	53	T4N0M0	10	CC	GT	GG	GG	
5	M	63	pT2aN0M0	0	CG	TT	GG	AG	
6	M	79	pT1aN0M0	30	CC	GG	CG	AG	
7	F	67	T3N3M0	20	CG	TT	CG	AA	
8	M	67	T2aN3M1b	0	CC	GT	CC	AA	
9	M	73	T2aN0M0	50	CC	GG	CC	AA	
10	F	78	T1cN1M0	0	CC	GT	GG	AA	
11	F	60	T1NM0	30	CG	GG	GG	AG	
12	F	68	T1cN2M0	50	CC	GG	CG	AA	
13	F	67	T2aN0M0	20	CG	GT	CG	AA	
14	M	70	T2aN0M0	50	CG	GT	CG	AA	
15	F	63	T1aN1M0	80	GG	TT	CC	AG	
16	F	68	T4N2M0	50	CC	GT	CG	AG	
17	M	70	T2bN0M0	20	CC	GT	CG	AA	
18	F	67	T2aN1M0	25	GG	TT	CG	AA	
19	M	77	T4NxM1b	30	CC	GT	GG	AG	
20	F	74	T2aN0M0	40	GG	GT	CG	AG	
21	F	68	T2bN2M0	30	CG	GG	GG	AA	
22	F	35	T1cN3M0	0	CC	GT	CG	AG	
23	F	68	T2aN1M0	50	CG	GG	CG	AA	
24	M	68	T1cN3M0	50	CG	GG	GG	AA	
25	M	67	T4N0M0	35	CC	GT	GG	AA	
26	M	78	T2aN0M0	0	CG	GG	GG	AG	
27	F	79	pT1cN0M0	0	CC	GT	CG	AA	
28	M	79	T2aN2M1a	60	GG	TT	CG	AA	
29	M	64	T1cN0M0	45	CC	GT	CG	AA	
30	M	73	T3N0M1	30	CC	GG	GG	AA	Adenocarcinoma (intestinal metastasis)
31	F	70	TxNxM1	0	CG	GT	CG	AA	
32	F	70	T1bN0M0	30	CG	GG	CG	AA	Squamous cell carcinoma
33	F	64	T2aN0M0	40	CC	GG	CG	AG	
34	M	65	T1cN0M0	50	CG	GG	CG	AA	
35	F	68	T2aN0M0	40	CG	GG	CG	AA	
36	M	68	T2aN0M0	50	CC	GT	CG	AA	
37	F	76	T2aN0M0	30	CC	GG	GG	AG	
38	F	71	T1cN1M0	5	CG	GT	GG	AA	
39	F	76	T1aN0M0	60	CC	GG	CG	AA	
40	F	77	T2aN0M0	10	GG	GT	GG	AA	
41	F	69	T1bN0M0	50	CG	GT	GG	AA	
42	F	71	T1cN0M0	50	CC	GG	CG	AA	
43	M	84	T3N3M0	10	CC	GT	CG	AG	
44	M	63	pT1bPL0N0M0	45	CC	GG	GG	AA	
45	M	70	T2aN0M0	30	CG	GT	CG	AA	
46	M	60	T3N1M0	30	CC	GG	CC	AA	
47	F	75	T2N2M0	30	CC	GG	CG	AA	
48	M	78	T2aN0M0	30	CG	GT	GG	AA	
49	F	74	T3N0M0	30	CG	GT	CG	AA	
50	F	82	T1bN0M0	0	CC	GG	CG	AA	

51	F	73	T3N0M0	0	CC	GG	CG	AA	
52	M	73	T2bN3M1c	60	CC	GG	CG	AA	
53	M	68	T4N0M0	45	CC	GG	CG	AA	
54	F	71	T2N2M0	25	CC	GG	GG	AA	Small-cell carcinoma
55	F	63	T1bN0M0	45	CG	GG	CC	AA	
56	F	70	T4N3M1c	50	GG	GG	CG	AA	
57	F	65	T4NxM1c	30	CC	GG	CG	AA	
58	F	65	T2bN0M0	30	CC	GG	CG	GG	
59	M	67	T4N2M0	50	CC	GG	CG	AA	
60	M	73	T1cN3M1b	40	CG	GG	GG	AG	
61	M	78	T2bN1M0	25	CC	GT	CG	AG	
62	F	75	T4N2M1c	50	CG	GT	CG	AA	
63	F	55	T4N3M1c	30	CC	GG	GG	AA	
